# Allometric scaling of the elevation of maternal energy intake during lactation

**DOI:** 10.1186/s12983-016-0164-y

**Published:** 2016-07-13

**Authors:** Frédéric Douhard, Jean-François Lemaître, Wendy M. Rauw, Nicolas C. Friggens

**Affiliations:** UMR Modélisation Systémique Appliquée aux Ruminants, Inra, AgroParisTech, Université Paris-Saclay, 75005 Paris, France; CNRS, UMR5558, Laboratoire de Biométrie et Biologie Evolutive, Université de Lyon, F-69000, Lyon, France – Université Lyon 1, F-69622 Villeurbanne, France; Departamento de Mejora Genética Animal, Instituto Nacional de Investigación y Tecnología Agraria y Alimentaria, Ctra. de La Coruña km 7, 28040 Madrid, Spain

**Keywords:** Energy intake, Lactation, Scaling, Reproductive effort

## Abstract

**Background:**

In most mammals, lactating mothers dramatically increase their food intake after parturition and reach a peak intake rate after a certain time while their offspring continue to grow. A common view, perpetuated by the metabolic theory of ecology, is that the allometric scaling of maternal metabolic rate with body mass limits the changes in energy intake and expenditure. Therefore these potential effects of metabolic scaling should be reflected in the elevation of maternal energy intake during lactation. To test this hypothesis, we collected published data on 24 species (13 domesticated) and established scaling relationships for several characteristics of the patterns of energy intake elevation (amplitude of the elevation, time to peak, and cumulative elevation to peak).

**Results:**

A curvilinear allometric scaling relationship with maternal body mass (in double-logarithmic space) was found for the amplitude of maternal energy intake elevation, similarly to what has been observed for scaling relationships of basal metabolic rate in non-breeding mammals. This result indirectly supports the metabolic theory of ecology. However, this curvilinear allometric scaling does not seem to drive the scaling relationships found for the other characteristics of maternal energy intake. Both the duration and shape of the energy intake patterns showed substantial variation independently of species’ body mass.

**Conclusions:**

Data available for a few mammals, mostly domesticated, provides little evidence for the hypothesis that a single law of metabolic scaling governs the elevation of maternal energy intake after parturition. Obtaining further food intake data in wild species will be crucial to unravel the general mechanisms underlying variation in this unique adaptation of mammalian females.

**Electronic supplementary material:**

The online version of this article (doi:10.1186/s12983-016-0164-y) contains supplementary material, which is available to authorized users.

## Background

It has long been recognized that large animals typically reproduce at a slower rate and live longer than small ones [1, 2], but the underlying physiological mechanism related to body size is still unclear. Besides, many biological characteristics ranging from cell to ecosystem level scale with body size, which led to the idea of a single size-dependent constraint on production, extensively developed in a metabolic theory of ecology (MTE) [3]. A common, yet controversial [4], viewpoint perpetuated by the MTE is that the allometric scaling of metabolic rate with body mass dictates the rate and duration of other biological processes, including key traits of mammalian reproduction [5–7]. In most of these studies, the energetic basis of reproductive allocation is often approached indirectly, using surrogate measures of maternal energy outputs (e.g., offspring mass, age at weaning, production rates [8–10]) and assuming that maternal energy inputs are extrinsically limited by the environment. In contrast, laboratory studies on mammalian energetics suggest that during lactation – the period of highest energy expenditure during reproduction [11–13] – the magnitude of a mother’s reproductive effort can be markedly affected by physiological limits to her energy intake [14–16]. Since the rate of maximal sustained energy intake scales allometrically with body mass [17, 18], the effects of metabolic scaling on a mother’s reproductive effort may be mediated through her energy acquisition.

**Fig. 1:**
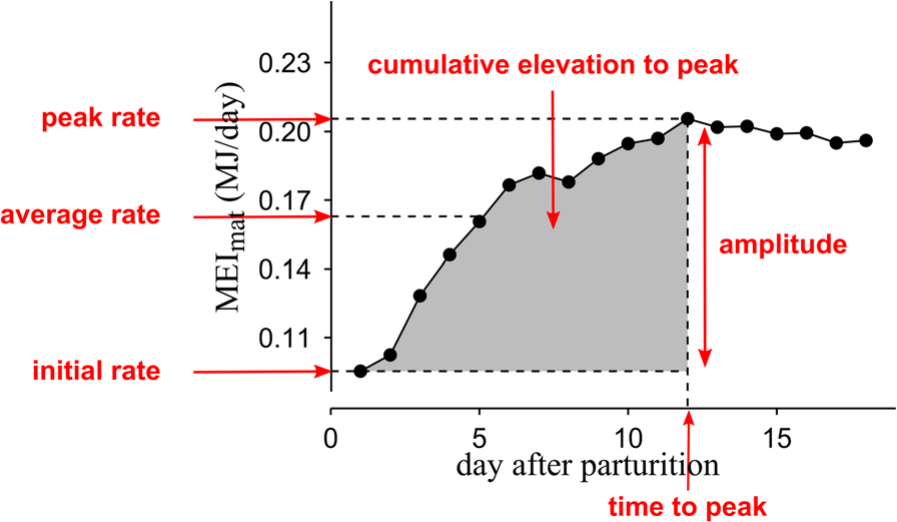
Example of a pattern of maternal metabolizable energy intake (MEI_mat_) elevation after parturition in lactatating Norway mice (*Mus musculus domesticus*). The main characteristics of the pattern are indicated with arrows. The MEI_mat_ is in mega-joule (MJ) per day. Data are from Rauw et al. [81]

Lactation involves numerous physiological changes [19], including a dramatic increase in the amount of energy that mothers consume and metabolize (MEI_mat_) after parturition [20, 21]. With the exception of some species that fast and rely mostly on stored body reserves during lactation (e.g., seals, bears) [22], most mammalian females elevate their MEI_mat_ up to a peak rate (Fig. 1), usually taken as a measure of their limiting capacity to acquire energy [20]. The decline of MEI_mat_ after the peak is most likely associated with the weaning process and a declining maternal contribution to offspring growth and viability [23]. The corresponding reproductive effort refers to the cumulative amount of energy committed to offspring [8, 24]. Therefore, in the context of MEI_mat_ elevation during lactation, it is of interest to compare the level of MEI_mat_ to the level observed under non-reproducing conditions [25], prior to conception. Unfortunately data on such comparisons are mainly reported in small rodents (e.g., [25]) and very rarely in larger mammals. Nevertheless, as shown in Fig. 1, the initial rate of MEI_mat_ at the onset of lactation can be used as a baseline, and on this basis the cumulative amount of energy consumed by mothers to support offspring growth and viability after birth can be calculated (i.e., cumulative elevation to peak; grey area in Fig. 1). Although the level of MEI_mat_ at the onset of lactation is higher than the level observed prior to conception, the elevation during gestation is relatively low compared to the elevation during lactation (e.g., +45 % *versus* +270 %, respectively, in mice [21]). It should also be noted that MEI_mat_ during gestation poorly reflects maternal effort to fetal development, possibly due to space competition between fetal mass and the alimentary tract within the abdomen [21].

The reproductive effort of female mammals has been suggested to vary proportionally to their body mass across the whole spectrum of species body sizes [26, 27]. Indeed, the rate-of-living theory [1, 2], posits that independently of species' body size, one gram of an organism’s tissue consumes and expends the same amount of food energy in its lifetime. Based on this, the MTE assumes that the product of the mass-specific metabolic rate and lifespan is constant regardless of body mass. Mathematically, if metabolic rate scales with body mass to the *b*-power, then lifespan scales with body mass to the (1 – *b*)-power, and so should most of the durations of biological events within life [3]. This latter prediction of the MTE has received less attention [4] compared to the debate about the scaling of metabolic rate itself (e.g., [28, 29]). However, recent studies in mammals have shown that after controlling for the phylogenetic relations between species, longevity [30] and the durations of reproductive events such as gestation [31] and lactation [32] generally present a scaling apparently unrelated to that of metabolic rate, and highly variable between taxonomic orders.

The expectation from the MTE that metabolic rate drives the rate and the duration of MEI_mat_ elevation during lactation can be translated in terms of scaling relationships with body mass, assuming that body mass is not the only but the most important factor determining metabolic rate in interspecific studies [33]. In particular, if metabolic rate only influences the rate and duration of MEI_mat_ elevation, only these two characteristics (and not the shape of MEI_mat_ patterns) would scale with body mass and determine the cumulative elevation to peak. For instance, if the shape of the patterns was uniformly linear, then the cumulative elevation to peak would be the surface area of a right-angled triangle (i.e., slightly less than observed in Fig. 1), that is half of the product between the time to peak and the amplitude of MEI_mat_ elevation. In that case only these two characteristics would depend on body mass and make up the cumulative elevation to peak of any species. In contrast, the shape can represent a third influencing characteristic if it includes enough variation among species (e.g., if mothers develop their MEI_mat_ more or less rapidly after parturition).

The MTE represents a valuable null theory to make a coherent set of hypotheses as it predicts scaling relationships with interrelated scaling exponents [34], but the fundamental principle of these interrelations (i.e., the existence of a single causal metabolic scaling law) may be too simplistic [4]. Here we test the following predictions: (*i*) the rate characteristics of the MEI_mat_ elevation of lactating mammal mothers (i.e., initial, peak, and average MEI_mat_) will scale with their body mass to the *b*-power, similarly to what is observed for basal metabolic rate. Since the log-log scaling of basal metabolic rate with body mass is apparently not linear (e.g., with a constant value of 3/4 for *b*) but curvilinear, concave-up, with *b* increasing from about 2/3 to more than 3/4 [29, 35–37], we expected similar changes in *b* with variation in maternal body mass, as proposed in [38] (*ii*) the biological time of the process of MEI_mat_ elevation (i.e., the time to peak) will scale with maternal body mass to the (1 – *b*)-power, and accordingly the scaling relationship should be curvilinear, concave-downward (*iii*) the cumulative elevation to peak of MEI_mat_ will scale isometrically with maternal body mass. In others words, over the whole lactation period of MEI_mat_ elevation, the cumulative elevation to peak will be proportional to the maternal body mass whether the mother is a small rodent or a large artiodactyl, and (*iv*) the shape of the patterns of MEI_mat_ elevation will be generally constant as a consequence of the three previous expectations.

To test the above predictions, and in particular to get reliable measures of the cumulative elevation to peak, data that fully describe the MEI_mat_ elevation from parturition to time to peak of a wide range of species were needed. Such data are only available for a few species, mainly laboratory or domestic animals, but with the advantage of being mostly free from ecological factors that can extrinsically limit MEI_mat_ (e.g., predation, resource shortage). Additionally, because mothers are fed *ad libitum* with a controlled diet, variation in diet energy density can be accounted for when analyzing energy intake. Although these data were difficult to find in a variety of different-sized species, they are well-suited for a focus on situations where an intrinsic limitation on energy intake may apply.

## Methods

### Dataset

We selected studies having three kinds of information: a complete description of the pattern of MEI_mat_ elevation during lactation (see Fig. 1), the energy density of the diet, and adult body mass. Energy intake was described on a daily basis, except for some studies in medium and large species which usually have a coarser description of food intake (weekly measurement). The observed lactation period had to be long enough to provide the two key points of Fig. 1: the initial rate – that is the first value of MEI_mat_ after the day of parturition (intake at parturition – that is day 0 – was not considered), and the peak rate – that is the maximum value of MEI_mat_ just before it tends to level off (avoiding potential outliers). The MEI_mat_ was expressed in mega-joule (MJ) of metabolizable energy (ME: gross energy in ingested food minus energy lost in feces, urine, and gases). Generally, studies included a single value of ME density for a given diet (in MJ/kg of dry matter), meaning that authors assumed a constant rate of energy assimilation during their experiment. In some studies, the energy density was not directly presented as ME so we used specific conversion equations. For instance, in a study of pigs (*Sus scrofa domesticus*) by Cooper et al. [39], the provided information on digestible energy density and chemical composition of the diet was used to predict the ME density with equations based on digestibility trials in the pig [40]. In most studies MEI_mat_ was calculated by multiplying the mass of food intake with the ME density of this food provided *ad libitum*. For instance in Sadleir [41], black-tailed hinds (*Odocoileus hemionus columbianus*) in their third week of lactation ate on average 2.65 kg/day of dry matter of a diet containing about 11.5 MJ of ME per kg of dry matter, therefore the corresponding MEI_mat_ was 30.5 MJ/day. The ME density of the diet can represent an extrinsic limitation on the amount of energy mothers can obtain and metabolize per day; when feeding animals with artificial diets, they typically increase diet intake with decreasing energy density but in lactating females this can be insufficient to offset the lower amount of ME per mass of food (e.g., [42]). Diet ME density was thus controlled in our statistical models used to establish scaling relationships. Scaling relationships are usually presented with adult body mass (log-transformed) as the independent variable. In the present study, body mass at the time of peak MEI_mat_ was used instead. Body mass changes during lactation but we assume that in most species the mass of active tissues in support of lactation (e.g., alimentary tract, mammary gland) is maximal at the time to peak so that it is an appropriate measure for comparisons. Most of the data were originally presented in figures and were digitalized using WebPlotDigitizer [43]. After an extensive literature search, we compiled a dataset comprising 52 patterns of MEI_mat_ representing 24 species (including eleven rodents, six artiodactyls, two lagomorphs, two carnivores, one perissodactyl, one primate, and one soricomorph) sometimes including different breeds (see Additional file 1 for the complete dataset, see Additional file 2: Table S1 and Fig. S1 for summarized information including phylogenetic tree of the represented species).

From each pattern representing the MEI_mat_ elevation during lactation we extracted four main characteristics (Fig. 1). The time to peak was the time after parturition (in days) when the peak rate was reached. The amplitude of MEI_mat_ elevation was calculated as the difference between the peak and the initial rate (in MJ/day). The average rate (in MJ/day), calculated from all extracted values between the initial and the peak rate, had no particular physiological meaning but was included to represent a MEI_mat_ during early- and mid-lactation more accurately than a single measurement. The cumulative elevation to peak MEI_mat_ (in MJ) was calculated as the sum of the differences between each MEI_mat_ measurement and the initial rate of MEI_mat_, from initial to peak rate.

### Variation in the shape of maternal energy intake patterns

To characterize the variations in the shape of MEI_mat_ patterns, we modelled the changes in the cumulative elevation of MEI_mat_ during lactation. The cumulative elevation of MEI_mat_ should be roughly proportional to the cumulative gain of offspring body mass, so we assumed that it could be modelled with a growth function. We applied the following modelling procedure using a generalized Von Bertalanffy growth equation [44]:

1 $$ cumulative\  elevation\ to\  time\ t=A\cdot {\left(1 - {e}^{-k\cdot t}\right)}^c, $$

where *t* is time after parturition (in days) and *k* the theoretical rate of MEI_mat_ elevation (in MJ/day). The asymptote *A* and parameter *c* do not have any biological meaning alone, but allow the derivation of three characteristics of MEI_mat_ patterns (i.e., cumulative elevation to peak, time to peak, and amplitude; see Additional file 2: Table S2). These three characteristics were linked through the parameter *c* as follows:

2 $$ cumulative\  elevation\ to\  peak= amplitude\cdot time\ to\  peak \cdot \frac{\left(1 - \frac{1}{c}\right)}{log(c)}\ . $$

The term $\frac{\left(1 - \frac{1}{c}\right)}{log(c)}$ was considered as a shape characteristic. This term would take value 0.5 if the MEI_mat_ elevation during lactation were perfectly linear because the cumulative elevation to peak would then be presented by the area under the curve of a right-angled triangle with length ‘time to peak’ and height ‘amplitude’ (Fig. 1). A shape value greater than 0.5 would increase the previous surface area, indicating that the pattern of MEI_mat_ elevation is concave downward (i.e., decelerating from parturition on) whereas a value lesser than 0.5 would decrease the surface area, indicating a concave up elevation of MEI_mat_ (i.e., accelerating from parturition on).

Equation (1) was adjusted to each pattern of cumulative elevation of MEI_mat_ using non-linear regressions and least-square estimates (nls function in R v. 3.0.2 [45]). The fitting procedure converged for all but two patterns of MEI_mat_ (those in the ground squirrel, *Callospermophilus lateralis,* and yellow baboon, *Papio cynocephalus).* The goodness-of-fit was evaluated for both the cumulative elevation and the elevation of MEI_mat_ using the root mean square error (RMSE) divided by the range of measured data (i.e., maximum – minimum) in each pattern. Finally, we checked for abnormal fits both visually and by detecting outliers of the relative difference between the time to peak estimated by the model and as defined in the original dataset. On this basis, three fitted patterns were excluded (including the only one for the deer mouse, *Peromyscus maniculatus*). So from the modelling procedure, MEI_mat_ characteristics were estimated for 47 patterns representing 21 species. Due to this reduction in the overall dataset, we analyzed the data using the raw measurements previously defined and using the model estimates, and we reported the results for both approaches.

### Scaling relationships

To analyze scaling relationships while accounting for the repeated measurements in species and breeds we used linear mixed models. The fixed effects included a linear term (*b*_*1*_) and a quadratic term (*b*_*2*_) for the log_10_-transformed maternal body mass, plus a term for the ME density of the diet (in MJ/kg of dry matter) and an intercept. Thus the assumed untransformed model had the form of a classic scaling relationship *y* = *a* ⋅ *x*^*b*^, where *y* is a characteristic of MEI_mat_ elevation and *x* of maternal body mass, but the normalization constant *a* was dependent on the diets’ ME density (*a* = 10^(intercept + diet ME density)^) and the scaling exponent *b* was dependent on body mass (*b* = *b*_*1*_ + *b*_*2*_∙log_10_*x*). Species, and breed nested within species were considered as random effects. For the species level, phylogenetic relationships were accounted for in our analyses. Indeed, the absence of phylogenetic correction when the phylogeny has a significant effect (or vice versa) can strongly bias estimations of the scaling exponent (e.g., [29–31]). In our phylogenetically-informed analyses, we determined for each scaling relationship the strength of a phylogenetic signal λ using Monte Carlo Markov chains in the R package MCMCglmm [46, 47] with phylogenetic information from the mammalian supertree of Bininda-Emonds et al. [48, 49]. The MCMC method allows the running of phylogenetic analysis with multiple measures per species. Thus all the available information on within-species variation (i.e., multiple patterns of MEI_mat_) could be directly considered in the analyses, without weighing effect, despite the heterogeneous species representation in our dataset (i.e., 28 of the 47 patterns of MEI_mat_ represented by 5 of the 24 species). The signal λ generally varies between 0 (i.e., no phylogenetic signal) and 1 (i.e., the observed pattern is predicted by the phylogeny) [50]. We used weakly informative priors for the random effects and ran the chains for 1,000,000 iterations (preceded by a burn-in of 15,000 iterations) and thinning interval of 100. The mean estimates of the scaling exponents from the posterior distribution along with the 95 % credible interval (CI) and the corresponding MCMC p-value were reported. To evaluate the influence of the phylogenetic structure of the data on the parameter estimation, results obtained with non-phylogenetically informed analyses in lme4 [51] were also reported.

Two sets of model fitting were performed. A first one included the raw measurements of the main characteristics of the MEI_mat_ patterns as reported in Fig. 1 (i.e., average rate, initial rate, peak rate, amplitude, time to peak, cumulative elevation to peak). Then we analyzed the characteristics estimated with the modelling procedure (as detailed previously) to determine any effect of the shape of MEI_mat_ patterns on the cumulative elevation to peak. For each regression, the goodness-of-fit was quantified with a marginal r^2^ that gives the variance explained by the fixed effects [52].

## Results

For most of the analyzed variables, the phylogenetic signal was relatively strong (λ between 0.6 and 0.8) although parameter estimation was relatively unchanged when the phylogenetic structure of the data was ignored (Table 1). Diet ME density had a significant positive effect on all the rate characteristics of MEI_mat_ except on the initial rate. Interestingly, when data were averaged per species and analyzed accordingly (i.e., with one point per species as commonly practiced), this effect of the diet ME density was no longer detected and slightly different allometric relationships (i.e., greater values of *b*_*2*_) were estimated (see Additional file 2: Table S3).

**Table 1:** Parameter estimates for the scaling of maternal metabolizable energy intake (MEI_mat_) during lactation established from the 24 species of the study, with and without accounting for the phylogenetic structure of the data

Characteristic of MEI_mat_ during lactation (log_10_)	Phylogeny controlled	Parameters				r^2^	λ
		Intercept	log_10_ body mass (*b*_*1*_)	log_10_ body mass^2^ (*b*_*2*_)	Diet energy density		
Average rate	Yes	−0.235* [−0.454, 0.005]	0.672*** [0.611, 0.728]	0.053** [0.024, 0.082]	0.018* [0.001, 0.033]	0.99	0.61
	No	−0.247* [−0.433, 0.060]	0.693*** [0.652, 0.733]	0.053*** [0.028, 0.078]	0.018* [0.004, 0.032]	0.99	–
Initial rate	Yes	−0.289 [−0.629, 0.082]	0.665*** [0.591, 0.739]	0.062** [0.023, 0.099]	0.006 [−0.019, 0.032]	0.99	0.03
	No	−0.288 [−0.310, −0.150]	0.673*** [0.616, 0.727]	0.064*** [0.033, 0.096]	0.005 [−0.018, 0.026]	0.99	–
Peak rate	Yes	−0.233 [−0.468, 0.010]	0.667*** [0.603, 0.732]	0.050** [0.019, 0.082]	0.024** [0.008, 0.040]	0.99	0.65
	No	−0.239* [−0.435, −0.041]	0.691*** [0.647, 0.736]	0.050*** [0.023, 0.078]	0.024** [0.009, 0.038]	0.99	–
Amplitude	Yes	−0.847*** [−1.317, −0.412]	0.643*** [0.531, 0.758]	0.055* [0.002, 0.110]	0.038* [0.008, 0.069]	0.98	0.74
	No	−0.807*** [−1.180, −0.400]	0.666*** [0.580, 0.750]	0.059* [0.010, 0.100]	0.037* [0.010, 0.070]	0.98	–
Time to peak	Yes	1.266*** [0.853, 1.659]	0.078 [−0.029, 0.185]	0.036 [−0.013, 0.085]	−0.001 [−0.028, 0.026]	0.52	0.79
	No	1.266*** [1.000, 1.400]	0.117 [0.041, 0.194]	0.034 [−0.011, 0.080]	−0.002 [−0.027, 0.022]	0.57	–
Cumulative elevation to peak	Yes	0.302 [−0.341, 0.947]	0.730*** [0.565, 0.910]	0.092* [0.013, 0.171]	0.032 [−0.012, 0.074]	0.96	0.81
	No	0.305 [−0.235, 0.893]	0.798*** [0.673, 0.922]	0.091* [0.017, 0.164]	0.033 [−0.011, 0.072]	0.97	–

λ is the strength of the phylogenetic signal (varying from 0 (absent) to 1 (strong))

Numbers in brackets are 95 % CI estimates

Asterisks denote the level of statistical significance: * (*P* < 0.05), ** (*P* < 0.01), *** (*P* < 0.001)

### Scaling relationships between characteristics of maternal energy intake

**Fig. 2:**
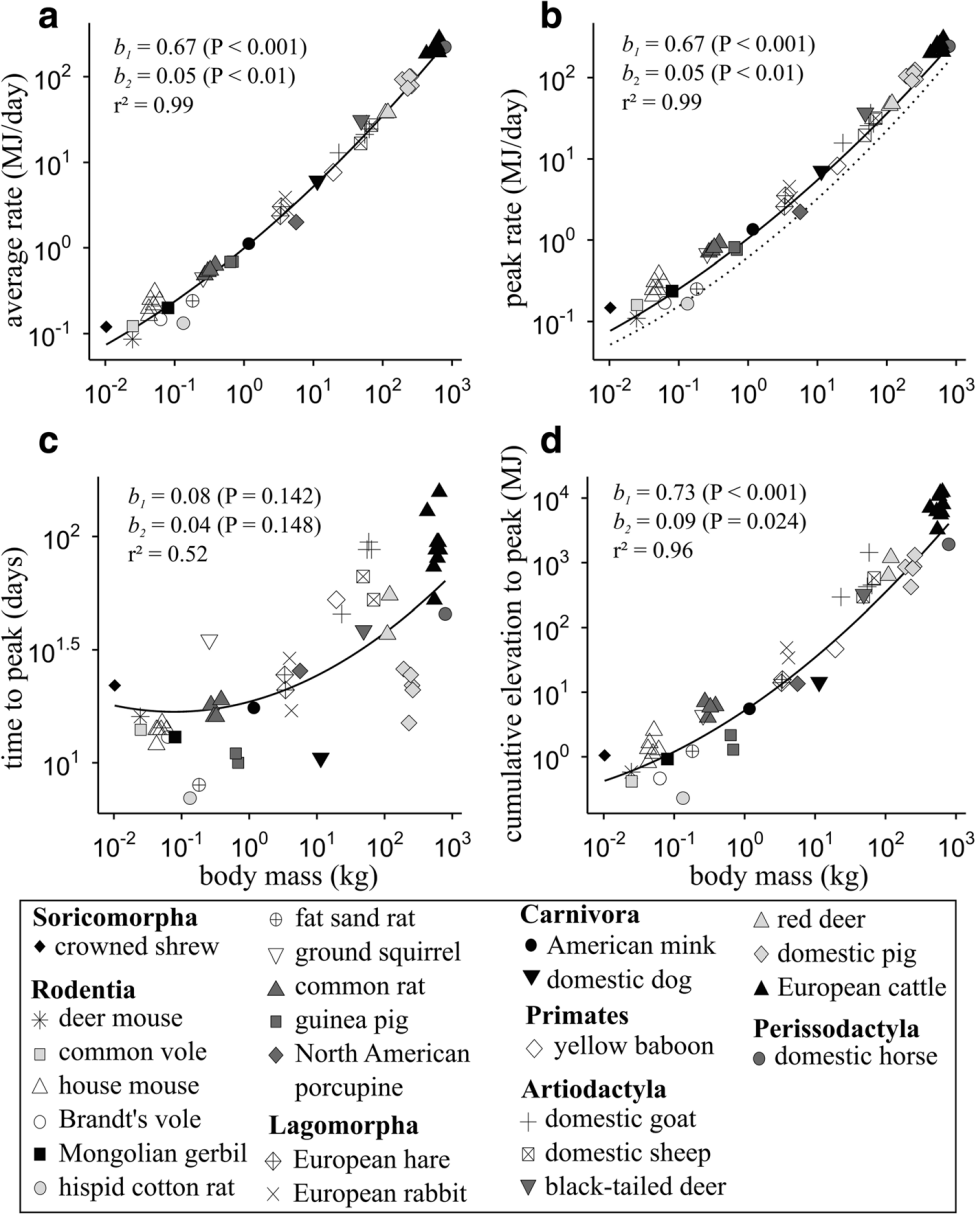
Log-log plots of the relation between maternal body mass and raw data of various characteristics of maternal metabolizable energy intake (MEI_mat,_ in MJ) elevation during lactation: (**a**) average rate of MEI_mat_ between parturition and time to peak, (**b**) peak rate (points and solid line) and initial rate (dotted line) of MEI_mat_, (**c**) time to peak, and (**d**) cumulative elevation to peak of MEI_mat_. For each characteristic, phylogenetically-informed analysis was applied to all data points (i.e., multiple measures per species) according to the regression model $y=a\cdot {x}^{\left({b}_1 + {b}_2\cdot { \log}_{10}x\right)}$, where coefficients *b*_*1*_ and *b*_*2*_ represent the linear and the quadratic effect, respectively, of maternal body mass in the log_10_-transformed model (log_10_ *y* = *b*_2_ ⋅ (log_10_ *x*)^2^ + *b*_1_ ⋅ log_10_ *x* + log_10_ *a*), and where the value of the normalization coefficient *a* depends on the diet energy density. Each panel includes the values of *b*_*1*_ and *b*_*2*_ (with their 95 % CI), their statistical significance, the fitted values of the phylogenetically-corrected model calculated at the average diet energy density (solid line), and the goodness-of-fit (marginal r^2^). Detailed model results are in Table 1

Figure 2 shows the raw measurements of the different MEI_mat_ characteristics during lactation in relation to maternal body mass. As expected, the average rate between parturition and the time to peak (Fig. 2a), the initial rate and the peak rate of MEI_mat_ (Fig. 2b) all scaled curvilinearily with maternal body mass in double-logarithmic space, and with relatively little variation around the regression lines (Table 1). This means that the value of the scaling exponent increased as mammals become larger; for instance for mothers around 50 g (like mice) the model predicted an exponent of 0.67 + 0.050 × log_10_(0.05) = 0.60 for the peak rate whereas for mothers around 500 kg (like cows) it rose to 0.80. The similarity of the parameters found for the amplitude of MEI_mat_ elevation (Table 1) shows that on average, independently of body mass, mothers increase their energy intake 1.9 times between the initiation of lactation and the time to peak (mean effect of the ratio peak rate/initial rate for which body mass effects *b*_*1*_ and *b*_*2*_ were not significant with *P* > 0.8). A non-significant curvilinear concave-up scaling relationship was observed for the time to peak (Fig. 2c), meaning that the scaling exponent tended to increase with maternal body mass instead of decreasing as we would expect from the MTE. Variation in the time to peak was loosely related to body mass differences. Interestingly, we observed that the two precocial rodent species in our dataset (i.e., hispid cotton rat, guinea pig) consistently reached their peak rate relatively early. However, the most important variations were found in larger species even intra-specifically (e.g., European cattle). Finally, contrary to our expectation, the scaling relationship observed for the cumulative elevation to peak of MEI_mat_ was not linear (isometric) but curvilinear (Fig. 2d). The degree of curvilinearity (*b*_*2*_ = 0.092) mostly reflected the sum of the effects of the squared logarithm of maternal body mass (*b*_*2*_) observed for the magnitude of MEI_mat_ elevation and for the time to peak (0.055 + 0.036 = 0.091, Table 1). The value of the scaling exponent estimated for the cumulative elevation to peak was close to the value of 1 predicted by the MTE in large mammals (e.g., 0.73 + 0.092 × log_10_(500) = 0.98 for mothers around 500 kg) but substantially less in small mammals (e.g., 0.61 for mothers around 50 g).

### Modelling of maternal energy intake patterns

**Fig. 3:**
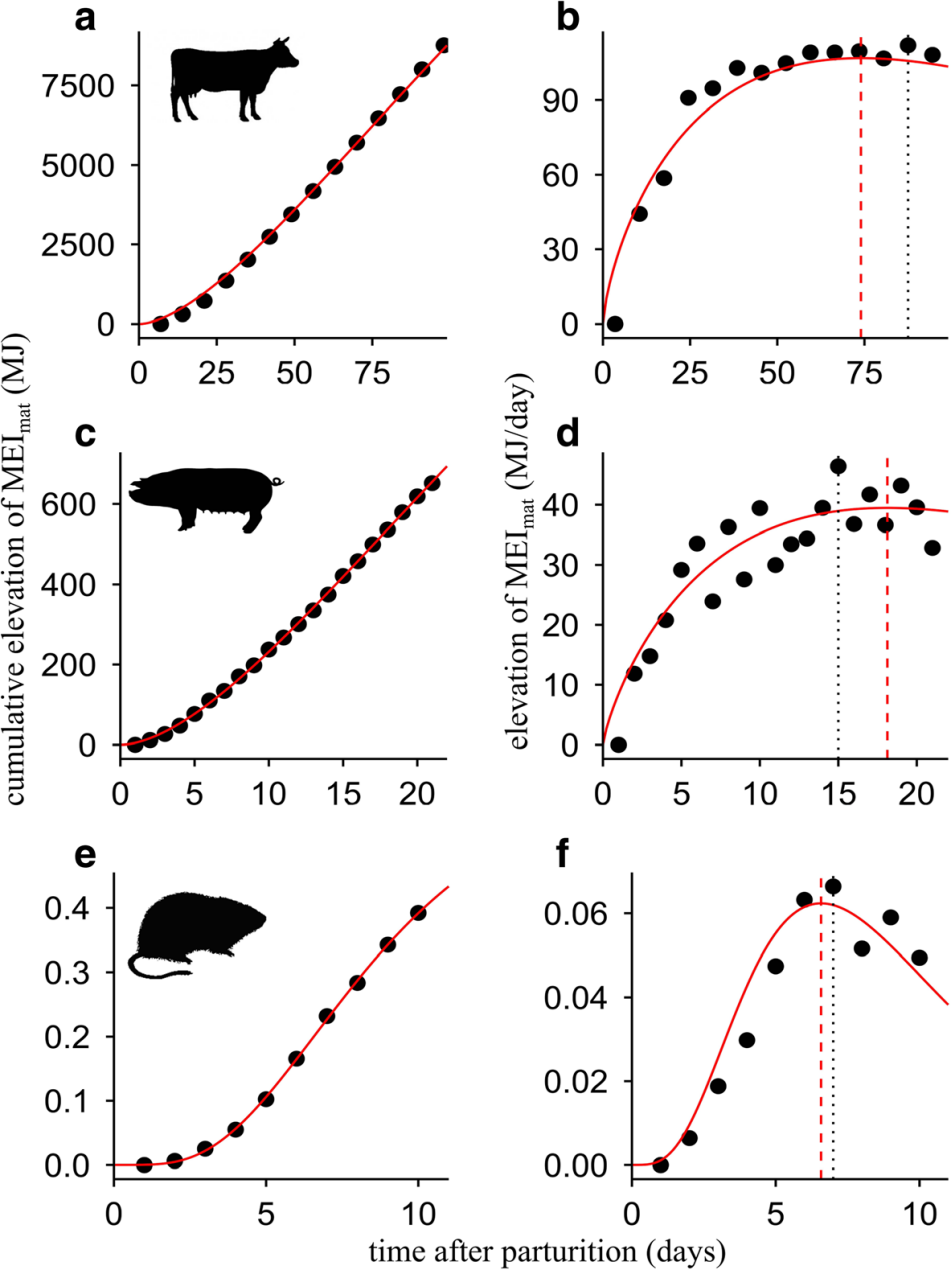
Examples of patterns of cumulative MEI_mat_ elevation fitted using Eq. (1) (left panels) and its derivate, i.e., the intake above the initial rate or extra intake (right panels). Points are observed values, lines are model fits for: **a**, **b** European dairy cattle (*Bos taurus taurus*), **c**, **d** domestic pig (*Sus scrofa domesticus*), **e**, **f** hispid cotton rat (*Sigmodon hispidus*). Values of the time to peak are reported both according to the modelling procedure (red dashed line) and as measured, i.e., when the peak rate of MEI_mat_ is observed (details in Material and Methods)

There was a high goodness-of-fit of the sigmoid model to each pattern of cumulative elevation of MEI_mat_ (normalized RMSE = 5.6 % on average). The accuracy was logically lower but fairly high for the rate of MEI_mat_ elevation (normalized RMSE = 9.6 % on average). The model was flexible enough to deal with different shapes of MEI_mat_ patterns (Fig. 3).

Scaling relationships established with the estimates from the modelling procedure exhibited exponents close to those previously obtained with the raw measurements (amplitude of MEI_mat_ elevation: *b*_*1*_ = 0.68, 95 % CI = 0.57 to 0.79, *P* < 0.001; *b*_*2*_ = 0.045, 95 % CI = − 0.010 to 0.100, *P* = 0.11; time to peak: *b*_*1*_ = 0.06, 95 % CI = − 0.07 to 0.19, *P* = 0.032; *b*_*2*_ = 0.049, 95 % CI = − 0.011 to 0.110, *P* = 0.011; cumulative elevation to peak:*b*_*1*_ = 0.74, 95 % CI = 0.52 to 0.96, *P* < 0.001; *b*_*2*_ = 0.093, 95 % CI = 0.012 to 0.191, *P* = 0.08). The slight differences were mainly due to the reduction in the dataset after the modelling procedure was applied and the 5 patterns of MEI_mat_ elevation without normal fitting were excluded (see Additional file 2: Table S5).

### Shape of maternal energy intake patterns

The shape parameter was, on average, greater than 0.5 (mean = 0.66, SE = 0.02), indicating that the increase of MEI_mat_ tends to decelerate from early lactation on with no initial acceleration phase. As illustrated in Fig. 3, the shape was variable (SD = 0.11). The variation among the 47 fitted MEI_mat_ patterns was completely independent of body mass (scaling exponent established for the shape = 0, 95 % CI = − 0.03 to 0.04). To investigate whether this variation explained residual variation in the cumulative elevation to peak of MEI_mat_, we included the shape parameter as a covariate in the scaling relationship. Logically we detected a positive effect of the shape on cumulative elevation to peak – the later mothers slowed down their increase in MEI_mat_ (i.e., the greater was the shape parameter), the higher was their cumulative elevation to peak MEI_mat_ (once the effects of body mass and diet ME density have been removed) – and this effect was significant (see Additional file 2: Table S5).

## Discussion

Results of this study indicate that in the light of recent published scaling relations of basal metabolic rate in mammals, the allometric scaling of energy intake in lactating mothers appears to be linked to their rate of metabolism. However, when examining interrelations between the scaling relationships established for several characteristics of the whole process of MEI_mat_ elevation during lactation (i.e., time to peak, cumulative elevation to peak, and shape of MEI_mat_ patterns), the view that metabolic scaling controls the changes in mothers’ energy consumption was no longer supported.

### Limitations of the study

Three kinds of potential limitations must be addressed in our analysis. Firstly, the relatively few number of species (*n* = 24) and the presence of a majority of domestic species (*n* = 13) in our dataset call for a cautious interpretation of our results in the context of the MTE. In particular, most of the large species were farm animals intensively selected for high energy expenditure (e.g., milk yield in the dairy cow, litter size in the sow) so their rate of MEI_mat_ might be higher than unselected animals. This could cause the curvature detected in the scaling relationship with body mass as pointed out in scaling studies of milk yield [53, 54]. Here, parameter estimates are relatively robust to the exclusion of one species or another from the dataset (e.g., predicted exponent of peak rate at 500 kg = 0.804, 0.806, 0.811, and 0.807, when excluding cows, sows, black-tailed deer, or mice, respectively). The relatively high energy consumption and expenditure of farm animals is also associated with a high ME density of the diet, an effect that was controlled for, and that was significantly positive in most of our results. Interestingly, this effect was no longer significant when data were averaged per species and were analyzed accordingly. Moreover, the very low variation around the regression lines for the rates of MEI_mat_ combined with the fact that artificial selection for high reproductive outputs in farm animals has substantially increased the level of body reserve mobilization in early lactation [55, 56], suggests that intake and digestive capacity are major factors limiting energy uptake in mammals. Yet, this suggestion must remain largely hypothetical until further data can be collected (especially in large wild mammals). Although our results seem consistent enough to discuss the profile of the scaling relationship, exact values of the estimated scaling exponents should be interpreted with caution.

Secondly, as we used maternal body mass at time to peak as the explanatory variable, this might cause a confounding effect with MEI_mat_ characteristics included as response variables. Indeed when MEI_mat_ increases during lactation, so does the mass of several maternal organs and tissues (e.g., mammary gland, alimentary tract), and so can the whole body mass compared to that observed under non-breeding conditions [54]. This generally occurs in mice for instance (e.g.,[57]). In contrast, in many species (e.g., common rat (*Rattus norvegicus*), dairy cow (*Bos taurus taurus*)) the mass loss due to the depletion of body reserve in support of lactation seems to predominate, leading to an overall decrease in body mass between the onset of lactation and the time to peak. In our dataset, the change in species’ body mass between the onset of lactation and the time to peak was mostly between – 10 and + 10 %, but establishing scaling relationships with maternal body mass at the onset of lactation did not markedly affect the value of the estimated scaling exponents (Additional file 2: Table S4).

A third issue relates to the use of MEI_mat_ at the onset of lactation as a baseline. As after birth mothers probably eat more than under non-breeding conditions, MEI_mat_ elevation during lactation could be underestimated. Moreover, this underestimation might have changed with body mass as in some large mammals food intake is strongly depressed during the first days after parturition [58]. So although our results indicate that in general maternal body size does not influence the scope to increase intake during lactation (i.e., the ratio peak intake/initial intake), this is not necessarily true when taking MEI_mat_ under non-breeding conditions as a baseline. From the limited information available, the ratio between MEI_mat_ of non-breeding females and the peak rate during lactation is not clearly independent of body size: 4.4 in mice [59], 2.8 in Mongolian gerbil [60], 2.2 in guinea pig [61], 1.9 in West African dwarf goat [62], 1.7 in black tailed deer [41]. Further, based on a rough MEI_mat_ estimation in non-breeding conditions (by applying a scaling equation of the field metabolic rate from [63] to our dataset), the scaling of the ratio peak rate/non-breeding rate of MEI_mat_ turned out to be slightly negative (*b* = − 0.064, *P* = 0.024). This implies that the scope to increase MEI_mat_ during lactation would decline as mammals get larger. All these indications suggest that establishing scaling equations based on data comparing breeding females to non-breeding females would be preferable, if sufficient of these data were available.

### Link between metabolic rate and the rate of maternal energy intake

Prior to this study, allometric scaling effects during mammalian lactation have been described for MEI_mat_ [14, 17, 18] and for different rates of expenditure (i.e., milk energy output: [19, 64]; milk yield:[53, 54, 64, 65]). These studies reported a value of the scaling exponent close to that historically found for the metabolic rate (i.e., between 2/3 and 3/4), but did not account for the shared ancestry between species nor did they allow the scaling exponent to vary with species’ body mass. When overcoming these limitations, the scaling exponent of the basal metabolic rate [36, 37] and of the field metabolic rate [63] showed a consistent increase with body mass from about 2/3 to more than 3/4, which corresponds to a curvilinear scaling pattern in double-logarithmic space. The present study indicates that such a scaling pattern also holds for the rates of MEI_mat_ (i.e., initial, average, and peak rates), so that a functional linkage between metabolic rate and energy intake cannot be excluded in lactating mammal mothers. Interestingly, Bueno and López-Urrutia found that a curvature exists for the scaling of ingestion rate and equally for that of other life-history traits from the organism level to the ecosystem level [38]. These findings, to some extent, provide support for the pervasive effect of metabolic scaling predicted by the MTE.

As the surface area and the volume of animals scales to the 2/3- and to the 1-power of body mass respectively, the decrease in the surface area to volume ratio with increasing body mass has been proposed as a proximate explanation for the change in *b* with body mass which causes the curvilinear scaling pattern of mammalian energy metabolism [37, 66]. The relatively large surface area of small mammals may cause a predominating influence of surface-related factors (e.g., heat loss) on their metabolic rate so *b* tends towards 2/3, whereas the metabolic rate of large mammals may be relatively more influenced by volume-related factors (e.g., tissue demand or resource transport network within the body) so *b* tends towards 1. Similarly, during lactation, a surface-related factor – the capacity to dissipate body heat [67, 68], and a volume-related factor – the demand of peripheral tissues, mainly the mammary glands [69], increasingly appear as the main factors limiting the rate of energy consumption and expenditure [16]. This combination of mechanisms may underlie both metabolic rate during resting and energy intake rate during lactation. This hypothesis seems more likely than the assumption from the MTE that body size limits the metabolic rate *via* the resource-transport network within the body, especially because an expansion of this network and an increase in blood flow to the mammary gland typically occur during lactation [66].

However, regardless of the factors underlying the scaling of the metabolic rate with body size, it is at best uncertain that metabolic scaling primarily causes the scaling relationships observed in energy intake and other processes. As discussed in [70], the scaling of metabolic rate changes with the level of metabolic activity (e.g., torpor, routine activity, strenuous exercise), so this may also happen during lactation – a metabolically intensive process. In the present study, although the different rates of MEI_mat_ during lactation (i.e., initial, average, and peak rates) had approximately the same scaling pattern, differences may be more obvious for measures of different physiological states (as previously discussed for the elevation of MEI_mat_ compared to non-breeding conditions). Interspecifically, a high correlation has been found between the amplitude of MEI_mat_ elevation during lactation, basal metabolic rate, and litter postnatal growth rate [25], but intraspecific studies show weak or no correlation between basal metabolic rate and metabolic rate during lactation [57] or reproductive output [71, 72]. Yet, basal metabolic rate responds to selection for food intake [73] and vice versa [74]. This suggests that a functional linkage exists between MEI_mat_ and metabolic rate but without a clear relationship of causality [4].

### Metabolic scaling and reproductive effort

Although our results are not completely in opposition to the main expectation from the MTE that metabolic scaling dictates the rate of MEI_mat_ during lactation, they more clearly contradict another expectation from this theory that metabolic scaling also drives the temporal changes and the whole pattern of MEI_mat_ elevation. Firstly, the scaling found for the time to peak did not coincide with the expectation of the MTE that it would be concave-downward (i.e. *b*_*2*_ was not negative). Secondly, this low scaling plus the substantial mass-independent variations in the patterns of MEI_mat_ elevation apparently contribute to a non-isometric scaling relationship of the cumulative elevation to peak with maternal body mass. A scaling exponent lower than 1 has been found for other cumulative proxy measures of reproductive effort [5, 8, 75], and this has been recently interpreted as a differential maternal energy allocation to reproduction along the spectrum of species body size [5]. The present study suggests that these differences result, at least partly, from a decreasing mass-specific energy intake over the lactation period as females get larger. However, the question remains as to whether the same result would be obtained if the elevation of MEI_mat_ was considered from conception onwards rather than from parturition. With respect to this, although several studies found a scaling exponent lower than the value of 1/4 predicted by the MTE for the scaling of gestation [31, 32] or lactation [32] duration, the sum of these two durations (i.e., development time) seems much closer to the MTE prediction in mammals [5], and primates [6]. Although the phylogenetically-informed analysis of Jackson et al. across mammals did not confirm this finding (*b* ≈ 0.15), except for primates [32]. Further research would be useful to compare the total maternal energy expenditure for offspring development, from conception to weaning, although this is technically challenging in particular for species where gestation and lactation overlap during successive reproductive events.

The non-significance of the curvilinear scaling pattern established for the time to peak was not necessarily related to the limited number of data included in the dataset. For instance, scaling of lactation duration (as approximated by age at weaning) established from much larger datasets also tends to produce wide variation among species independently of body mass [5, 8, 32]. Across four main orders of mammals, Jackson et al. [32] reported a scaling exponent with a confidence interval of 0.15 to 0.23. Such variation may reflect the effects of pup energy demand on maternal metabolism. For instance, increasing the energy demand of the mammary gland through experimental enlargement of litter size in mice [76], exposing the pups to the cold in hares [77], or frequent milking in dairy cows [78], all seem to accelerate the MEI_mat_ elevation, thus increasing the concavity of the pattern. In the present study, similar effects were partly indicated by greater values of our shape parameter which was independent of body size and significantly contributed to the cumulative elevation to peak. This concurs with the view that size-independent variation in production rate represents an axis for differentiating lifestyles (i.e., a suite of interrelated traits reflecting adaptations of lineages to their ecological conditions) [10]. Most of the species in our dataset have evolved specialized lifestyles on abundant and reliable foods (e.g., grazing and browsing herbivores). Therefore, obtaining data for mammals with contrasted lifestyles (e.g., insectivores) would be necessary to investigate to what extent the maternal ability to rapidly reach peak energy intake is part of differentiating lifestyles.

To what extent mothers can respond to pup energy demand may indeed reflect lineage-specific adaptations to ecological conditions (e.g., nutrition, predation) [10]. For instance, the relatively short time to peak we observed for the domestic pig corroborates the life history of its wild boar ancestor, which is characterized by unusually high and early reproductive effort compared to similar-sized artiodactyls (i.e., early primiparity, large litter size [79]). This characteristic might have been accentuated in the domestic pig due to intense selection for large litter size in the breeding industry, but most likely represents some typical adaptation of the fast life-history of the Suidae family, like their early decline of survival with age or their high litter size [79]. From a broader perspective, Müller et al. [63] have shown that in mammals, monotocous species (i.e., where females produce a single pup per litter) are generally larger and have a steeper scaling of metabolic rate than polytocous species (i.e., when females produce multiple pups per litter). They further showed that establishing two linear scaling relationships of metabolic rate – one for monotocous and one for polytocous – captures most of the variation explained by an overall curvilinear scaling pattern. It would be interesting to investigate whether or not this dichotomy has the same effects on the scaling patterns that we found for the characteristics of MEI_mat_, if more data were available. As the elevation and the sustainment of metabolism of lactating mothers entails various physiological costs that potentially impair their survival [21], characteristics of MEI_mat_ elevation might represent essential evolutionary adaptations [77]. These ultimate factors are in line with the assimilation capacity hypothesis [80] which, conversely to the MTE, proposes that an increase metabolic rate occurs as a correlated response to selection for a high rate of energy processing favoring offspring growth and survival.

## Conclusions

To conclude, we acknowledge the value of the MTE as a principle to unify various scaling relationships under one framework [34]. However, our results based on data available for relatively few mammals, mostly domesticated species, challenge the assumption of a single causal metabolic scaling law during mammalian lactation. Thus the validity of the MTE cannot be deduced only from the coincidence between the curvature of metabolic scaling and that found for a single aspect of a biological process (e.g., rates of MEI_mat_ during lactation). Alternatively, we proposed to consider simultaneously the multiple aspects of a process (e.g., duration and cumulative characteristics of MEI_mat_ elevation) to test the MTE as a unifying principle. Several advantages of collecting repeated measurements on the same set of individuals have been pointed out recently to make reliable comparisons between scaling relationships (i.e., lower incidence of intraspecific variability and experimental error) [38]. Concerning energy intake, repeated measures are presently available for a few, mostly domesticated mammals which limit the generalization of our results. Future data acquisition in a range of wild species will be precious to better understand the link between physiological limits on energy metabolism and the diversity of mammalian reproductive strategies.

## Additional files

Additional file 1: Data used in this study. Information on the patterns of MEI_mat_ during lactation is summarized (Sheet 'Study summary') and reported in details with all the points (Sheet 'Study whole data'). (XLSX 119 kb) Additional file 2: Additional files including a summary of the dataset used in this study (Table S1) with phylogenetic relationships between represented species (Figure S1), the list of equations used to model each pattern of MEI_mat_ elevation during lactation (Table S2), and results of additional analyses (Table S3-S5). (DOCX 266 kb)
