# Supplementary material for: Allometric scaling of the elevation of maternal energy intake during lactation
Source: Front Zool. 2016 Jul 13;13:32. doi: 10.1186/s12983-016-0164-y (PMC4944469; doi:10.1186/s12983-016-0164-y)
Supplement: Additional file 2: — Additional files including a summary of the dataset used in this study (Table S1) with phylogenetic relationships between represented species (Figure S1), the list of equations used to model each pattern of MEImat elevation during lactation (Table S2), and results of additional analyses (Table S3-S5). (DOCX 266 kb) [file 12983_2016_164_MOESM2_ESM.docx]

**Additional file**

**Allometric scaling of the elevation of maternal energy intake during lactation**, Frontiers in Zoology, Frédéric Douhard^1^, Jean-François Lemaître, Wendy M. Rauw, and Nicolas C. Friggens

^1^Author for correspondence; adress: Modélisation Systémique Appliquée aux Ruminants, Inra, AgroParisTech, Université Paris-Saclay, 75005, Paris, France;

Email: douhard@agroparistech.fr

**Table S1** Summary of the dataset used in the study (sorted by body mass observed at peak rate of maternal energy intake during lactation), with *n* the number of observed animals

| Species | Breed^[[1]](#footnote-1)^ | *n* | body mass (kg) | Reference |
| --- | --- | --- | --- | --- |
| \| \| Crowned shrew  Deer mouse \| \| --- \| \| Common vole \| \| House mouse \| \| House mouse \| \| House mouse \| \| House mouse \| \| House mouse \| \| House mouse \| \| Brandt's vole \| \| Mongolian gerbil \| \| Hispid cotton rat  Fat sand rat \| \| Ground squirrel \| \| Common rat \| \| Common rat \| \| Common rat \| \| Common rat \| \| Guinea pig \| \| Guinea pig \| \| American mink \| \| European hare \| \| European hare \| \| European rabbit \| \| European rabbit  North american porcupine \| \| Domestic dog  Yellow baboon \| \| Domestic goat \| \| Domestic dog \| \| Domestic sheep  Black-tailed deer \| \| Domestic goat \| \| Domestic goat \| \| Domestic goat \| \| Domestic sheep \| \| Red deer \| \| Red deer \| \| Domestic pig \| \| Domestic pig \| \| Domestic pig \| \| Domestic pig \| \| Domestic pig \| \| European cattle \| \| European cattle \| \| European cattle \| \| European cattle \| \| European cattle \| \| European cattle \| \| European cattle \| \| European cattle \| \| European cattle \| \| Domestic horse \| \| \| --- \| --- \| --- \| --- \| --- \| --- \| --- \| --- \| --- \| --- \| --- \| --- \| --- \| --- \| --- \| --- \| --- \| --- \| --- \| --- \| --- \| --- \| --- \| --- \| --- \| --- \| --- \| --- \| --- \| --- \| --- \| --- \| --- \| --- \| --- \| --- \| --- \| --- \| --- \| --- \| --- \| --- \| --- \| --- \| --- \| --- \| --- \| --- \| --- \| | \| N/A  N/A \| \| --- \| \| N/A \| \| MF1 \| \| Norwegian \| \| MF1 \| \| Swiss \| \| ICR \| \| Norwegian \| \| N/A \| \| N/A \| \| N/A  N/A \| \| Golden-Mantled \| \| Sprague-Dawley \| \| Lister \| \| Wistar \| \| Long-Evans \| \| N/A \| \| N/A \| \| N/A \| \| N/A \| \| N/A \| \| Provisal \| \| Chinchilla  N/A \| \| Beagle  N/A \| \| West African Dwarf \| \| Labrador Retriever \| \| Sarde  N/A \| \| Saanen \| \| Saanen \| \| Saanen x Alpine \| \| Scottish Blackface \| \| Scottish \| \| Scottish \| \| Swedish Landrace x Yorshire \| \| Large White \| \| Camborough 15 \| \| Landrace x Large White \| \| N/A \| \| Jersey \| \| British-Friesian x Ayrshire \| \| Holstein-Friesian \| \| British-Friesian x Ayrshire \| \| Holstein-Friesian \| \| Holstein-Friesian \| \| Danish red \| \| Holstein-Friesian \| \| Holstein-Friesian \| \| Breton x Comtois \| | \| 15  5 \| \| --- \| \| 16 \| \| 54 \| \| 81 \| \| 71 \| \| 25 \| \| 6 \| \| 94 \| \| 23 \| \| 11 \| \| 16  15 \| \| 18 \| \| 5 \| \| 14 \| \| 12 \| \| 11 \| \| 9 \| \| 22 \| \| 4 \| \| 13 \| \| 28 \| \| 12 \| \| 10  3 \| \| 5  6 \| \| 10 \| \| 17 \| \| 19  6 \| \| 4 \| \| 4 \| \| 9 \| \| 12 \| \| 4 \| \| 4 \| \| 30 \| \| 18 \| \| 72 \| \| 20 \| \| 51 \| \| 31 \| \| 8 \| \| 10 \| \| 8 \| \| 23 \| \| 34 \| \| 29 \| \| 11 \| \| 10 \| \| 5 \| | \| 0.010  0.025 \| \| --- \| \| 0.025 \| \| 0.043 \| \| 0.043 \| \| 0.045 \| \| 0.050 \| \| 0.052 \| \| 0.060 \| \| 0.063 \| \| 0.080 \| \| 0.133  0.181 \| \| 0.257 \| \| 0.272 \| \| 0.300 \| \| 0.325 \| \| 0.385 \| \| 0.634 \| \| 0.687 \| \| 1.170 \| \| 3.343 \| \| 3.434 \| \| 3.939 \| \| 4.204  5.630 \| \| 11.41  19.33 \| \| 23.29 \| \| 30.26 \| \| 48.08  49.00 \| \| 55.07 \| \| 58.44 \| \| 64.00 \| \| 68.90 \| \| 109.9 \| \| 118.9 \| \| 191.2 \| \| 229.0 \| \| 237.0 \| \| 254.6 \| \| 260.4 \| \| 427.3 \| \| 530.7 \| \| 543.9 \| \| 573.2 \| \| 598.8 \| \| 604.0 \| \| 627.3 \| \| 642.5 \| \| 651.5 \| \| 787.0 \| | \| [1]  [2] \| \| --- \| \| [3] \| \| [4] \| \| [5] \| \| [6] \| \| [7] \| \| [8] \| \| [9] \| \| [10] \| \| [11] \| \| [12]  [13] \| \| [14] \| \| [15] \| \| [16] \| \| [17] \| \| [18] \| \| [19] \| \| [20] \| \| [21] \| \| [22] \| \| [23] \| \| [24] \| \| [25]  [26] \| \| [27]  [28] \| \| [29] \| \| [30] \| \| [31]  [32] \| \| [33] \| \| [33] \| \| [34] \| \| [35] \| \| [36] \| \| [36] \| \| [37] \| \| [38] \| \| [39] \| \| [40] \| \| [41] \| \| [42] \| \| [43] \| \| [44] \| \| [43] \| \| [45] \| \| [42] \| \| [42] \| \| [46] \| \| [46] \| \| [47] \| |

**
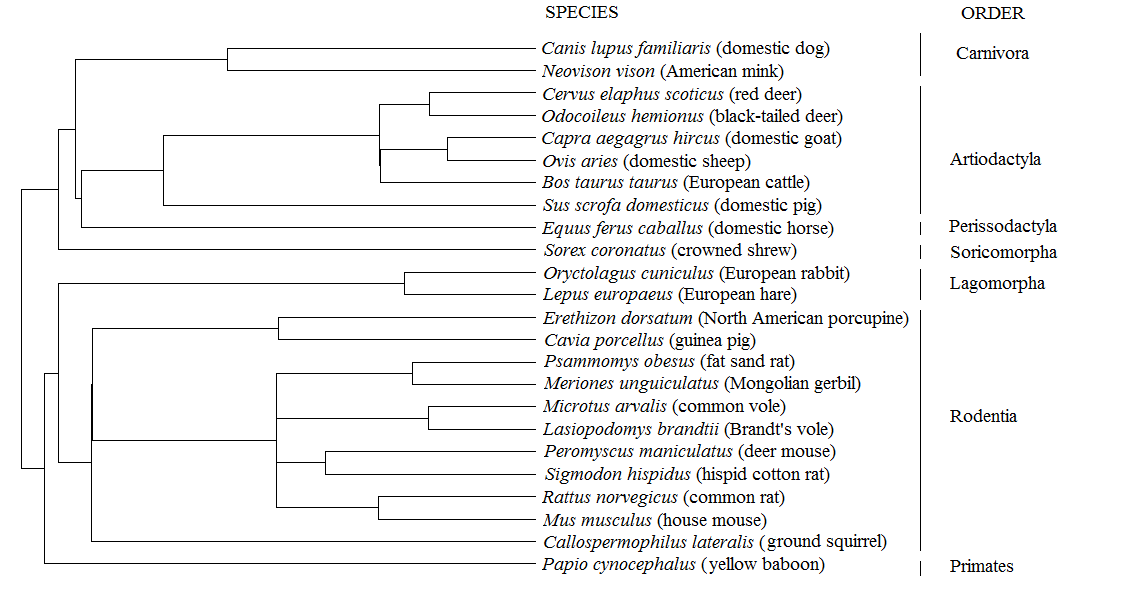
 Fig. S1** Phylogenetic tree for the 24 species included in the dataset

**Table S2** List of equations used to estimate the characteristics of maternal metabolizable energy intake (MEI_mat_) elevation during lactation with the model fitting procedure. Parameter *A* is a theoretical asymptote of cumulative elevation to peak, *t* is the time after parturition (in days), *k* the theoretical rate of MEI_mat_ elevation (MJ/day), *c* a scaling exponent

| Estimated variable or characteristic  of MEI_mat_ elevation during lactation | Equation |
| --- | --- |
| cumulative elevation to time *t* (in MJ) | $=A{\cdot\left( 1- e^{-k\cdot t} \right)}^{c}$ |
| elevation to time *t* (in MJ/day) | = $\frac{d(cumulative elevation)}{dt}$ |
|  | $=A{\cdot c\cdot k\cdot\left( 1- e^{-k\cdot t} \right)}^{c-1}\cdot e^{-k\cdot t}$ |
| time to peak (in days) | = $\frac{log(c)}{k}$ |
| cumulative elevation to peak (in MJ) | = A${\cdot\left( 1-\frac{1}{c} \right)}^{c}$ |
| amplitude of MEI_mat_ elevation (in MJ/day) | = A${\cdot k\cdot\left( 1-\frac{1}{c} \right)}^{c-1}$ |

**Table S3** Parameter estimates for the scaling of maternal metabolizable energy intake (MEI_mat_) during lactation established from the 24 species of the dataset with the multiple measures per species or with one average measure per species

| characteristic of MEI_mat_ during lactation (log_10_) | analyzed dataset | parameters | | | | r² | λ |
| --- | --- | --- | --- | --- | --- | --- | --- |
|  |  | intercept | log_10_ body  mass (*b_1_*) | log_10_ body mass² (*b_2_*) | diet energy density |  |  |
| average rate | multiple | -0.235*  [-0.454, 0.005] | 0.672***  [0.611, 0.728] | 0.053**  [0.024, 0.082] | 0.018*  [0.001, 0.033] | 0.99 | 0.61 |
|  | averaged | -0.240  [-0.551, 0.057] | 0.668***  [0.603, 0.735] | 0.063***  [0.033, 0.095] | 0.016  [-0.004, 0.038] | 0.99 | 0.11 |
| initial rate | multiple | -0.289  [-0.629, 0.082] | 0.665***  [0.591, 0.739] | 0.062**  [0.023, 0.099] | 0.006  [-0.019, 0.032] | 0.99 | 0.03 |
|  | averaged | -0.450**  [-0.799, -0.129] | 0.682***  [0.614, 0.750] | 0.060**  [0.026, 0.093] | 0.019  [-0.004, 0.043] | 0.99 | 0.07 |
| peak rate | multiple | -0.233  [-0.468, 0.010] | 0.667***  [0.603, 0.732] | 0.050**  [0.019, 0.082] | 0.024**  [0.008, 0.040] | 0.99 | 0.65 |
|  | averaged | -0.230  [-0.556, 0.108] | 0.663***  [0.593, 0.630] | 0.061**  [0.028, 0.095] | 0.022  [-0.001, 0.046] | 0.99 | 0.08 |
| amplitude | multiple | -0.847***  [-1.317, -0.412] | 0.643***  [0.531, 0.758] | 0.055*  [0.002, 0.110] | 0.038*  [0.008, 0.069] | 0.98 | 0.74 |
|  | averaged | -0.640*  [-1.200, -0.059] | 0.621***  [0.499, 0.737] | 0.069*  [0.014, 0.128] | 0.021  [-0.019, 0.060] | 0.97 | 0.94 |
| time to peak | multiple | 1.266***  [0.853, 1.659] | 0.078  [-0.029, 0.185] | 0.036  [-0.013, 0.085] | -0.001  [-0.028, 0.026] | 0.52 | 0.79 |
|  | averaged | 1.287***  [0.742, 1.827] | 0.071  [-0.042, 0.185] | 0.036  [-0.015, 0.091] | -0.002  [-0.038, 0.037] | 0.40 | 0.91 |
| cumulative elevation to peak | multiple | 0.302  [-0.341, 0.947] | 0.730***  [0.565, 0.910] | 0.092*  [0.013, 0.171] | 0.032  [-0.012, 0.074] | 0.96 | 0.81 |
|  | averaged | 0.635  [-0.185, 1.490] | 0.693***  [0.496, 0.865] | 0.108*  [0.023, 0.192] | 0.006  [-0.053, 0.061] | 0.95 | 0.98 |

λ is the strength of the phylogenetic signal (varying from 0 (absent) to 1 (strong))

asterisks denote the level of statistical significance: * (P < 0.05), ** (P < 0.01), *** (P < 0.001)

numbers in brackets are 95% CI estimates

**Table S4** Parameter estimates (with 95% CIs) for the scaling of the characteristics of maternal metabolizable energy intake (MEI_mat_) elevation during lactation (raw measures) with maternal body mass observed at the onset of lactation of at time to peak. The log_10_-transformed body mass, its square, and the metabolizable energy content of the diet (in MJ/kg of dry matter) were considered as fixed effects whereas species, and breed nested within species were considered as random effects. For the species level, phylogenetic relationships were accounted for in our analyses by using phylogenetically controlled analysis in MCMCglmm [48] (i.e. analyses correcting for the non-independence of species due to shared ancestries) to compute the strength of the phylogenetic signal λ (varying from 0 (absent) to 1 (strong)).

|  | analyzed data | model fitted body mass at the onset of lactation | | | | | | model fitted with body mass at time to peak | | | | | |
| --- | --- | --- | --- | --- | --- | --- | --- | --- | --- | --- | --- | --- | --- |
| characteristic of MEI_mat_ during lactation | parameter | post mean | 95% CI | | p-value | λ | r² | post mean | 95% CI | | p-value | λ | r² |
| log_10_ average rate | intercept  log_10_ body mass (*b_1_*)  log_10_ body mass² (*b_2_*)  diet energy density | – 0.270  0.661  0.063  0.018 | – 0.517  0.598  0.032  0.001 | – 0.028  0.719  0.092  0.036 | 0.032  < 0.001  < 0.001  0.037 | 0.09 | 0.99 | – 0.262  0.669  0.057  0.019 | – 0.501  0.608  0.028  0.003 | – 0.030  0.727  0.087  0.036 | 0.029  < 0.001  0.002  0.020 | 0.61 | 0.99 |
| log_10_ initial rate | intercept  log_10_ body mass (*b_1_*)  log_10_ body mass² (*b_2_*)  diet energy density | –0.330  0.648  0.073  0.007 | – 0.670  0.574  0.034  – 0.018 | 0.060  0.718  0.108  0.034 | 0.072  < 0.001  0.001  0.592 | 0.04 | 0.99 | – 0.329  0.659  0.066  0.008 | – 0.689  0.586  0.030  - 0.019 | – 0.039  0.732  0.106  0.034 | 0.080  < 0.001  0.002  0.530 | 0.03 | 0.99 |
| log_10_ peak rate | intercept  log_10_ body mass (*b_1_*)  log_10_ body mass² (*b_2_*)  diet energy density | – 0.257  0.656  0.060  0.024 | – 0.529  0.592  0.027  0.006 | 0.001  0.722  0.092  0.042 | 0.057  < 0.001  0.001  0.013 | 0.07 | 0.99 | – 0.250  0.665  0.054  0.024 | –0.495  0.601  0.022  0.008 | 0.003  0.729  0.085  0.041 | 0.047  < 0.001  0.003  0.005 | 0.69 | 0.99 |

**Table S4** (next)

|  | analyzed data | model fitted with body mass at the onset of lactation | | | | | | model fitted with body mass at time to peak | | | | | |
| --- | --- | --- | --- | --- | --- | --- | --- | --- | --- | --- | --- | --- | --- |
| characteristic of MEI_mat_ during lactation | parameter | post mean | 95% CI | | p-value | λ | r² | post mean | 95% CI | | p-value | λ | r² |
| log_10_ amplitude | intercept  log_10_ body mass (*b_1_*)  log_10_ body mass² (*b_2_*)  diet energy density | – 0.793  0.627  0.064  0.034 | – 1.288  0.506  0.005  – 0.001 | – 0.294  0.744  0.121  0.067 | 0.001  < 0.001  0.041  0.050 | 0.70 | 0.97 | – 0.815  0.641  0.058  0.036 | – 1.299  0.527  0.003  0.004 | – 0.355  0.757  0.115  0.067 | 0.002  < 0.001  0.045  0.028 | 0.78 | 0.98 |
| log_10_ time to peak | intercept  log_10_ body mass (*b_1_*)  log_10_ body mass² (*b_2_*)  diet energy density | 1.237  0.079  0.036  0.001 | 0.796  – 0.027  – 0.013  – 0.029 | 1.670  0.186  0.087  0.029 | < 0.001  0.141  0.144  0.944 | 0.73 | 0.53 | 1.245  0.081  0.034  0.001 | 0.822  – 0.027  – 0.017  – 0.029 | 1.687  – 0.192  – 0.085  – 0.029 | < 0.001  0.144  0.191  0.946 | 0.72 | 0.52 |
| log_10_ cumulative elevation to peak | intercept  log_10_ body mass (*b_1_*)  log_10_ body mass² (*b_2_*)  diet energy density | 0.315  0.718  0.098  0.030 | – 0.368  0.536  0.012  – 0.020 | 1.100  0.896  0.182  0.078 | 0.349  < 0.001  0.027  0.216 | 0.77 | 0.93 | 0.298  0.731  0.093  0.032 | – 0.410  0.560  0.010  – 0.016 | 1.008  0.911  0.175  0.078 | 0.410  < 0.001  0.031  0.179 | 0.81 | 0.96 |

**Table S5** Parameter estimates (with 95% CIs) for the scaling of the characteristics of maternal energy intake (MEI) elevation during lactation, obtained using a modelling procedure for each individual pattern or simply using raw estimates as in Table S2. Due to no-convergence and abnormal fits the initial dataset was reduced to 47 patterns representing 21 species. Scaling relationships were phylogenetically controlled as described in Table S4

|  | analyzed data | estimates of the modelling procedure | | | | | | raw measures in the reduced dataset | | | | | |
| --- | --- | --- | --- | --- | --- | --- | --- | --- | --- | --- | --- | --- | --- |
| characteristic of MEI_mat_ during lactation | parameter | post mean | 95% CI | | p-value | λ | r² | post mean | 95% CI | | p-value | λ | r² |
| log_10_ amplitude | intercept  log_10_ body mass (*b_1_*)  log_10_ body mass² (*b_2_*)  diet energy density | – 0.912  0.680  0.045  0.046 | – 1.364  0.565  – 0.010  0.016 | – 0.481  0.792  0.100  0.077 | < 0.001  < 0.001  0.108  0.003 | 0.64 | 0.98 | – 0.871  0.676  0.049  0.043 | – 1.346  0.554  – 0.010  0.013 | – 0.439  0.794  0.104  0.075 | < 0.001  < 0.001  0.098  0.008 | 0.69 | 0.98 |
| log_10_ time to peak | intercept  log_10_ body mass (*b_1_*)  log_10_ body mass² (*b_2_*)  diet energy density | 1.204  0.064  0.049  – 0.004 | 0.771  – 0.067  – 0.011  – 0.034 | 1.666  0.192  0.110  0.026 | < 0.001  0.324  0.106  0.781 | 0.83 | 0.44 | 1.212  0.077  0.041  – 0.002 | 0.778  – 0.037  – 0.011  – 0.029 | 1.608  0.186  0.091  0.026 | < 0.001  0.172  0.112  0.910 | 0.73 | 0.55 |
| log_10_ cumulative elevation to peak | intercept  log_10_ body mass (*b_1_*)  log_10_ body mass² (*b_2_*)  diet energy density | 0.077  0.739  0.093  0.040 | – 0.679  0.518  – 0.012  – 0.007 | 0.824  0.964  0.191  0.091 | 0.840  < 0.001  0.082  0.106 | 0.92 | 0.95 | 0.203  0.762  0.081  0.040 | – 0.501  0.566  – 0.010  – 0.008 | 0.918  0.962  0.168  0.086 | 0.555  < 0.001  0.074  0.102 | 0.85 | 0.96 |

**Table S5** (next)

|  | analyzed data | estimates from the modelling procedure | | | | | | raw measures in the reduced dataset | | | | | |
| --- | --- | --- | --- | --- | --- | --- | --- | --- | --- | --- | --- | --- | --- |
| characteristic of MEI_mat_ during lactation | parameter | post mean | 95% CI | | p-value | λ | r² | post mean | 95% CI | | p-value | λ | r² |
| log_10_ cumulative elevation to peak | intercept  log_10_ body mass (*b_1_*)  log_10_ body mass² (*b_2_*)  diet energy density  shape | – 0.959  0.754  0.069  0.063  1.264 | – 1.796  0.559  – 0.024  0.018  0.602 | – 0.147  0.947  0.154  0.103  1.951 | 0.027  < 0.001  0.129  0.007  0.001 | 0.87 | 0.99 | – 0.711  0.777  0.061  0.059  1.112 | – 1.584  0.600  – 0.019  0.017  0.407 | 0.104  0.946  0.143  0.103  1.851 | 0.096  < 0.001  0.128  0.010  0.002 | 0.79 | 0.99 |

**References in additional file**

1. Genoud, M. & Vogel, P. 1990 Energy requirements during reproduction and reproductive effort in shrews ( Soricidae ). *J. Zool.* **220**, 41–60.

2. Koteja, P. 1996 Limits to the energy budget in a rodent, Peromyscus maniculatus: the central limitation hypothesis. *Physiol. Zool.* **69**, 981–993.

3. Simons, M. J. P., Reimert, I., van der Vinne, V., Hambly, C., Vaanholt, L. M., Speakman, J. R. & Gerkema, M. P. 2011 Ambient temperature shapes reproductive output during pregnancy and lactation in the common vole (Microtus arvalis): a test of the heat dissipation limit theory. *J. Exp. Biol.* **214**, 38–49.

4. Vaanholt, L. M., Sinclair, R. E. & Speakman, J. R. 2013 Limits to sustained energy intake. XIV. Heritability of reproductive performance in mice. *J. Exp. Biol.* **216**, 2308–15.

5. Rauw, W. M., Knap, P. W., Varona, L. & Noguera, J. L. 2003 Reallocation of body resources in lactating mice highly selected for litter size. *J. Anim. Sci.* **81**, 939–944.

6. Johnson, M. S., Thomson, S. C. & Speakman, J. R. 2001 Limits to sustained energy intake. III. Effects of concurrent pregnancy and lactation in Mus Musculus. *J. Exp. Biol.* **1956**, 1947–1956.

7. Zhao, Z.-J., Chi, Q.-S. & Cao, J. 2010 Milk energy output during peak lactation in shaved Swiss mice. *Physiol. Behav.* **101**, 59–66.

8. Schubert, K. A., de Vries, G., Vaanholt, L. M., Meijer, H. A. J., Daan, S. & Verhulst, S. 2009 Maternal energy allocation to offspring increases with environmental quality in house mice. *Am. Nat.* **173**, 831–40.

9. Rauw, W. M. 2001 Changes in food resource allocation patterns with selection for litter size - a mouse model.

10. Wu, S.-H., Zhang, L.-N., Speakman, J. R. & Wang, D.-H. 2009 Limits to sustained energy intake. XI. A test of the heat dissipation limitation hypothesis in lactating Brandt’s voles (Lasiopodomys brandtii). *J. Exp. Biol.* **212**, 3455–65.

11. Yang, D.-B., Li, L., Wang, L.-P., Chi, Q.-S., Hambly, C., Wang, D.-H. & Speakman, J. R. 2013 Limits to sustained energy intake. XIX. A test of the heat dissipation limitation hypothesis in Mongolian gerbils (Meriones unguiculatus). *J. Exp. Biol.* **216**, 3358–68.

12. Randolph, P. A., Mattingly, K. & Foster, M. M. 1977 Energy cost of reproduction in the cotton rat, Sigmodon Hispidus. *Ecology* **58**, 31–45.

13. Kam, M. & Degen, A. A. 1993 Energetics of lactation and growth in the fat sand rat, Psammomys obesus: new perspectives of resource partitioning and the effect of litter size. *J. Theor. Biol.* **162**, 353–369.

14. Kenagy, G. J., Stevenson, R. D. & Masman, D. 1989 Energy requirements for lactation and postnatal growth in captive golden-mantled ground squirrels. *Physiol. Zool.* **62**, 470–487.

15. Barnett, M. P. G., Phillips, A. R. J., Harris, P. M. & Cooper, G. J. S. 2008 Impaired insulin secretion in perfused pancreases isolated from offspring of female rats fed a low protein whey-based diet. *J. Pancreas* **9**, 477–488.

16. Rolls, B. J. & Rowe, E. A. 1982 Pregnancy and lactation in the obese rat: effects on maternal and pup weights. *Physiol. Behav.* **28**, 393–400.

17. Stocker, C. et al. 2004 Modulation of susceptibility to weight gain and insulin resistance in low birthweight rats by treatment of their mothers with leptin during pregnancy and lactation. *Int. J. Obes. Relat. Metab. Disord.* **28**, 129–36.

18. Künkele, J. & Kenagy, G. J. 1997 Inefficiency of lactation in primiparous rats: the cost of first reproduction. *Physiol. Zool.* **70**, 571–577.

19. Künkele, J. & Trillmich, F. 1997 Are precocial young cheaper? Lactation energetics in the guinea pig. *Physiol. Zool.* **70**, 589–596.

20. Künkele, J. 2000 Energetics of gestation relative to lactation in a precocial rodent, the guinea pig (Cavia porcellus). *J. Zool.* **250**, 533–539.

21. Fink, R., Tauson, A. H., Chwalibog, A. & Hansen, N. E. 2006 A first estimate of the amino acid requirement for milk production of the high-producing female mink (Mustela vison). *J. Anim. Physiol. Anim. Nutr. (Berl).* **90**, 60–69.

22. Hackländer, K., Tataruch, F. & Ruf, T. 2002 The effect of dietary fat content on lactation energetics in the European hare (Lepus europaeus). *Physiol. Biochem. Zool.* **75**, 19–28.

23. Valencak, T. G. & Ruf, T. 2009 Energy turnover in European hares is centrally limited during early, but not during peak lactation. *J. Comp. Physiol. B.* **179**, 933–943.

24. Xiccato, G., Bernardini, M., Castellini, C. & Zotte, A. D. 1999 Effect of postweaning feeding on the performance and energy balance of female rabbits at different physiological states. *J. Anim. Sci.* **77**, 416–426.

25. Martínez-Gómez, M., Juárez, M., Distel, H. & Hudson, R. 2004 Overlapping litters and reproductive performance in the domestic rabbit. *Physiol. Behav.* **82**, 629–36.

26. Farrell, B. C. & Christian, D. P. 1987 Energy and water requirements of lactation in the North American porcupine, Erethizon dorsatum. *Comp. Biochem. Physiol. A. Comp. Physiol.* **88**, 695–700.

27. Romsos, D. R., Palmer, H. J., Muiruri, K. L. & Bennink, M. R. 1981 Influence of a low carbohydrate diet on performance of pregnant and lactating dogs. *J. Nutr.* **111**, 678–689.

28. Roberts, S., Cole, T. & Coward, W. 1985 Lactational performance in relation to energy-intake in the baboon. *Am. J. Clin. Nutr.* **41**, 1270–1276.

29. Adenuga, M. K., Tolkamp, B. J., Ademosun, A. A., Montsma, G. & Brouwer, B. O. 1991 Effect of pregnancy and lactation on liveweight, feed intake and feeding behavior in West African Dwarf (WAD) goats. *Small Rumin. Res.* **4**, 245–255.

30. Legrand-Defretin, V. & Munday, H. S. 1993 Feeding dogs and cats for life. In *The Waltham Book of Companion Animal Nu trition (Burger, I. ed)*, pp. 57–68. Pergamon, Oxford, U.K.:

31. Bocquier, F., Ligios, F., Molle, G. & Casu, S. 1997 Effet de la photopériode sur la production , la composition du lait et sur les consommations volontaires chez la brebis laitière. *Ann. Zootech.* **46**, 427–438.

32. Sadleir, R. M. F. S. 1982 Energy consumption and subsequent partitioning in lactating black-tailed deer. *Can. J. Zool.* **60**, 382–386.

33. Zambom, M. A., Alcade, C. R., Martins, E. N., Dos Santos, G. T., Fonseca de Macedo, F. A., Horst, J. A. & Da Veiga, D. R. 2005 Lactation curve and milk quality of Saanen goats fed diets with different forage:concentrate ratios. *Rev. Bras. Zootec.* **34**, 2515–2521.

34. Schmidely, P. In press. unpublihshed data.

35. Foot, J. Z. & Russel, A. J. F. 1979 The relationship in ewes between voluntary food intake during pregnancy and forage intake during lactation and after weaning. *Anim. Prod.* **28**, 25–39.

36. Asher, G. W., Stevens, D. R., Archer, J. A., Barrell, G. K., Scott, I. C., Ward, J. F. & Littlejohn, R. P. 2011 Energy and protein as nutritional drivers of lactation and calf growth of farmed red deer. *Livest. Sci.* **140**, 8–16.

37. Neil, M. 1996 Ad libitum lactation feeding of sows introduced immediately before, at, or after farrowing. *Anim. Sci.* **63**, 497–505.

38. Prunier, A., De Braganqa, M. M. & Le Dividich, J. 1997 Influence of high ambient temperature on performance of reproductive sows. *Livest. Prod. Sci.* **52**, 123–133.

39. Cooper, D. R., Patience, J. F., Zijlstra, R. T. & Rademacher, M. 2001 Effect of nutrient intake in lactation on sow performance : determining the threonine requirement of the high-producing lactating sow. *J. Anim. Sci.* **79**, 2378–2387.

40. Mosnier, E., Etienne, M., Ramaekers, P. & Père, M. C. 2010 The metabolic status during the peri partum period affects the voluntary feed intake and the metabolism of the lactating multiparous sow. *Livest. Sci.* **127**, 127–136.

41. Kiefer, C., Martins, L. P. & Fantini, C. C. 2012 Evaporative cooling for lactating sows under high ambient temperature. *Rev. Bras. Zootec.* **41**, 1180–1185.

42. Nielsen, H. M., Friggens, N. C., Jensen, J. & Ingvartsen, K. L. 2003 Influence of breed, parity, and stage of lactation on lactational performance and relationship between body fatness and live weight. *Livest. Prod. Sci.* **79**, 119–133.

43. Garnsworthy, P. C. & Topps, J. H. 1982 The effect of body condition of dairy cows at calving on their food intake and performance when given complete diets. *Anim. Prod.* **35**, 113–119.

44. Bar-Pelled, U. et al. 1995 Relationship between frequent milking or suckling in early lactation and milk production of high producing dairy cows. *J. Dairy Sci.* **78**, 2726–2736.

45. Douglas, G. N., Overton, T. R., Bateman, H. G., Dann, H. M. & Drackley, J. K. 2006 Prepartal plane of nutrition, regardless of dietary energy source, affects periparturient metabolism and dry matter intake in Holstein cows. *J. Dairy Sci.* **89**, 2141–2157.

46. Friggens, N. C., Emmans, G. C., Kyriazakis, I., Oldham, J. D. & Lewis, M. 1998 Feed intake relative to stage of lactation for dairy cows consuming total mixed diets with a high or low ratio of concentrate to forage. *J. Dairy Sci.* **81**, 2228–2239.

47. Doreau, M., Boulot, S., Bauchart, D., Barlet, J.-P. & Martin-Rosset, W. 1992 Voluntary intake, milk production and plasma metabolites in nursing mares fed two different diets. *J. Nutr.* **122**, 992–999.

48. Hadfield, J. D. 2010 MCMC methods for multi-response generalized linear mixed models: The MCMCglmm R package. *J. Stat. Softw.* **33**, 1–22.

49. Bates, D., Maechler, M., Bolker, B., Walker, S. & Christensen, RHB Singmann, H. 2014 lme4: linear mixed-effects models using Eigen and S4.

1. N/A: non applicable [↑](#footnote-ref-1)
